# Magnetic Resonance Imaging in Uremic Encephalopathy: Identifying Key Imaging Patterns and Clinical Correlations

**DOI:** 10.3390/jcm13144092

**Published:** 2024-07-12

**Authors:** Federico Greco, Andrea Buoso, Laura Cea, Valerio D’Andrea, Caterina Bernetti, Bruno Beomonte Zobel, Carlo Augusto Mallio

**Affiliations:** 1 Department of Radiology, Cittadella della Salute, Azienda Sanitaria Locale di Lecce, Piazza Filippo Bottazzi, 2, 73100 Lecce, Italy; 2 Research Unit of Radiology, Department of Medicine and Surgery, Università Campus Bio-Medico di Roma, Via Alvaro del Portillo, 21, 00128 Roma, Italy; andrea.buoso@unicampus.it (A.B.); laura.cea@unicampus.it (L.C.); valerio.dandrea@unicampus.it (V.D.); c.bernetti@policlinicocampus.it (C.B.); c.mallio@policlinicocampus.it (C.A.M.); 3 Fondazione Policlinico Universitario Campus Bio-Medico, Via Alvaro del Portillo, 200, 00128 Roma, Italy

**Keywords:** uremic encephalopathy, renal failure, uremia, toxic and metabolic encephalopathies, basal ganglia, magnetic resonance imaging

## Abstract

**Background/Objectives:** Magnetic Resonance Imaging (MRI) is essential in diagnosing neurological conditions, offering detailed insights into brain pathology. Uremic encephalopathy (UE) is a severe neurological disorder resulting from renal failure, characterized by cognitive impairments and brain abnormalities due to the accumulation of uremic toxins (UTs). Despite extensive research on UTs, there is a significant gap in the detailed characterization of MRI findings in UE patients. This study aims to bridge this gap by conducting a comprehensive literature review of cerebral MRI findings in UE. We hypothesize that specific MRI patterns correlate with the severity and clinical manifestations of UE, thereby enhancing diagnostic accuracy and improving patient outcomes. **Methods:** A literature review was performed using PubMed, Cochrane Library, and Google Scholar. The search terms included “uremic encephalopathy MRI”, “uremia and kidney failure MRI”, and “toxic and metabolic or acquired encephalopathies MRI”. The inclusion criteria were original articles on UE and MRI findings published in English. **Results:** Common MRI sequences include T1-weighted, T2-weighted, FLAIR, and DWI. Frequent MRI findings in UE are cytotoxic and vasogenic brain edema in regions such as the basal ganglia and periventricular white matter. Patterns like the “lentiform fork sign” and basal ganglia involvement are key indicators of UE. **Conclusions:** MRI plays a crucial role in diagnosing UE by identifying characteristic brain edema and specific patterns. A comprehensive diagnostic approach, incorporating clinical, laboratory, and imaging data, is essential for accurate diagnosis and management. The study calls for larger well-designed cohorts with long-term follow-up to improve the understanding and treatment of UE.

## 1. Introduction

Magnetic Resonance Imaging (MRI) is a non-invasive imaging technique widely utilized in medical diagnostics to produce detailed images of the internal structures of the body [1]. Its ability to provide high-resolution images makes it particularly valuable in evaluating neurological conditions [1]. MRI is considered the modality of choice for assessing brain pathology, offering critical insights into structural changes and abnormalities that might not be detectable through other imaging techniques [1]. This imaging technology is crucial in diagnosing conditions such as uremic encephalopathy (UE), where subtle and specific changes in the brain structure need to be identified accurately.

UE is an acute neurological condition arising from the accumulation of toxic substances in the bloodstream, particularly urea and other protein metabolism products, due to acute or chronic severe renal failure [2,3,4]. These uremic toxins (UTs), including indoxyl sulfate and para-cresol sulfate, are potent inducers of oxidative stress, which can lead to cerebrovascular disease, cognitive disorders, and dementia. UTs contribute to vascular dysfunction, endothelial cell dysfunction, neuro-inflammation, blood–brain barrier disruption, glial cell activation, and neuronal death [5,6,7,8,9,10,11,12]. Clinical cognitive manifestations of UE usually are fluctuating and can vary depending on the rate of decline in renal function and, hence, eGFR (Table 1). In fact, accumulation of UTs resulting from acute renal failure leads to severe cognitive changes when the eGFR falls below 15 mL/min, whereas mild to moderate cognitive changes can be identified with higher eGFR values [3,13,14]. Patients undergoing haemodialysis may develop uremic encephalopathy if their treatments are insufficient for various reasons, including noncompliance or dysfunction of the arteriovenous fistula [15,16].

Diagnosis can be difficult, and it is often made retrospectively because of the many confounding and overlapping conditions seen in patients with chronic kidney disease and acute kidney injury. Furthermore, there is no specific laboratory test to confirm or diagnose UE [17]. Despite the extensive research on the effects of UTs and their role in UE pathogenesis, there is a significant gap in the detailed characterization of MRI findings in UE patients. Previous studies have documented cognitive changes associated with UTs accumulation, but comprehensive analyses correlating these changes with specific MRI findings are limited [6,7,8,9,10,11,12]. This gap underscores the need for more precise investigations to aid in the diagnosis and management of UE using MRI. The current literature lacks a thorough synthesis of MRI findings that can guide clinicians and radiologists in identifying and interpreting brain abnormalities in UE patients accurately.

The objective of this study is to perform a comprehensive literature review of cerebral MRI findings in UE, aiming to bridge the gap between past research and present diagnostic needs. We hypothesize that specific MRI patterns correlate with the severity and clinical manifestations of UE, which can significantly enhance the diagnostic accuracy and improve patient outcomes. By analyzing existing MRI studies on UE, this review seeks to provide a detailed reference for clinicians and radiologists, facilitating better diagnostic and therapeutic strategies for this complex condition.

## 2. Material and Methods

### 2.1. Search Strategy

We performed a comprehensive literature review by searching multiple online databases including PubMed, Cochrane Library, and Google Scholar. Our aim was to identify all relevant articles on the topic of UE and the corresponding MRI findings. The search terms included: “uremic encephalopathy MRI”, “uremia and kidney failure MRI”, “uremia MRI”, and “toxic and metabolic or acquired encephalopathies MRI”.

The inclusion criteria were publication in English of original articles pertaining to UE; there was no date limit. Exclusion criteria encompassed publications not available in English and studies that did not use the MRI technique.

### 2.2. Data Extraction

The following information was extracted: author and year of publication, country, number of study participants, rationale of the study, and the study results.

We summarize the main characteristics of the MRI studies focused on UE in Table 2.

## 3. The Role of MRI in UE

Imaging plays a crucial role in the assessment of UE, particularly through the use of MRI which is favored over computed tomography due to its superior ability to detail the extent and nature of brain involvement, aiding in the differential diagnosis and management of the condition.

MRI sequence protocols include T1-weighted imaging (T1WI), T2-weighted imaging (T2WI), and fluid-attenuated inversion recovery (FLAIR) sequences, which can provide valuable information about the tissue characteristics, lesions, and potential complications. Other useful sequences are diffusion-weighted imaging (DWI), which could be useful to discriminate vasogenic edema from cytotoxic edema and, hence, determine whether the symptoms are caused by ischemia or by cerebrovascular autoregulatory dysfunction [23,24].

Contrast-enhanced MRI usually has a marginal role, mostly to evaluate the presence of associated vascular abnormalities or inflammatory processes.

The most common imaging finding described in the literature is cytotoxic and/or vasogenic brain edema in the basal ganglia (BG), internal capsules, and periventricular white matter, as well as in the frontal, parietal and occipital cortex [23,24,25,26].

Edema in the involved cerebral areas presents as areas of low signal intensities on T1WI, high signal intensities on T2WI/FLAIR, with or without restricted diffusion (cytotoxic vs. vasogenic) [18].

There are three main patterns of localization described in the literature: cortical or subcortical areas, BG, and white matter [18,27].

Several studies demonstrated how the BG is a region vulnerable to toxins and metabolic changes, such as UTs accumulation in renal failure, for instance, in diabetic patients [28].

Wang et al. [19] explored the relationship between BG involvement at MRI and neurological manifestations, underlining the importance of considering uremia as a possible cause of acute movement disorders, especially in patients with advanced chronic kidney disease or undergoing dialysis treatment. Notably, bilateral lesions of the BG in uremia could often manifest with acute and subacute extrapyramidal movement disorders, such as dyskinesias, asterixis, myoclonus, and, less frequently, with seizures. These findings suggest that the syndrome of acute bilateral BG lesions can be a significant neurological manifestation in diabetic uremic patients [19,20,21].

In addition, Lee et al. [20] observed that acute bilateral BG lesions can occur in patients with uremia, specifically diabetic, as demonstrated especially by DWI. The observed hyperintensity on DWI and hypointensity on ADC maps suggest cytotoxic edema as the underlying pathophysiological mechanism. These results and conclusions underscore the importance of DWI in identifying and understanding neurological complications in diabetic patients with uremia, particularly those involving acute BG lesions, representing a valuable tool for detecting and characterizing them [20].

**Figure 1:**
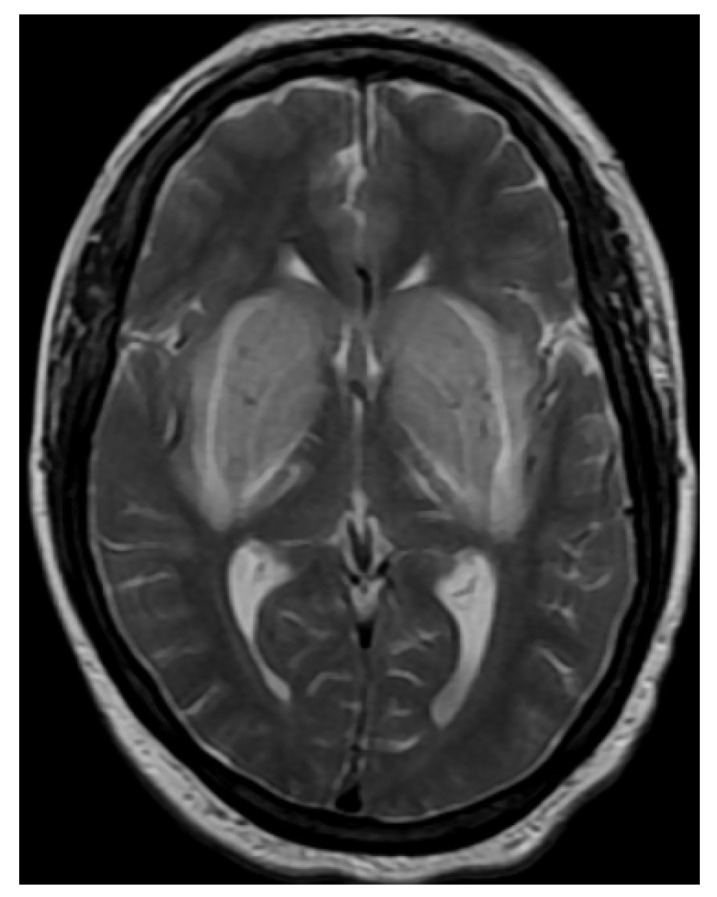
T2WI image on the axial plane shows bilateral swollen and edematous lentiform nuclei, presenting as hyperintense lesions. These lesions display three hyperintense lines, corresponding to the edematous external capsule and the external and internal medullary laminae, dividing the lentiform nucleus into the putamen, globus pallidus externa, and interna from lateral to medial, creating the characteristic ‘lentiform fork sign’. Reproduced from ref. [29], under the Creative Commons Attribution-NonCommercial-ShareAlike 4.0 International License.

A larger retrospective study conducted by Kim et al. [18] investigated the MR imaging findings and their correlation with clinical symptoms in patients with UE, revealing various abnormalities in the brains, including changes in the BG, white matter, and cerebral cortex. MR imaging showed bilateral, expansile, symmetric BG lesions with increased signal intensity on T2WI, FLAIR, and/or ADC maps, compatible with vasogenic edema. One of the key MRI indicators of UE detected was the “lentiform fork sign” (LFS), which is characterized by a high signal intensity in the BG on T2WI (Figure 1) [18,29]. Furthermore, this sign was found as a reliable marker in the early stages of the condition [18,30]. The emergence of the LFS is believed to be closely associated with metabolic acidosis and the subsequent disruption of the blood–brain barrier [31,32]. However, in this study, metabolic acidosis was observed in only one patient with the LFS [18]. This finding suggested that the presence of the LFS may not be solely dependent on the occurrence of metabolic acidosis. Instead, this study proposed that the LFS can manifest in UE regardless of whether metabolic acidosis is present.

Recently, Sina et al. [22] evaluated the diagnostic utility of MRI in the identification of UE. Through an in-depth review of MRI findings, including identification of specific patterns such as white matter involvement and brain atrophy, along with comprehensive clinical assessments and laboratory analysis, the researchers aimed to clarify whether MRI could offer a definitive diagnosis of UE [22].

The study evaluated BG involvement, white matter damage, the presence of the LFS, manifestations similar to posterior reversible encephalopathy syndrome (PRES), and cortical or subcortical atrophy. Specifically, it was noted that among the prospectively reviewed patients with UE, none showed involvement of the BG or the LFS. However, a significant proportion, around two-thirds of the patients, exhibited white matter involvement, while 80% of them displayed cerebral or cortical atrophy on MRI scans. Additionally, arterial blood gas analysis indicated that half of the patients experienced metabolic acidosis. Further, a significant relationship was found between PRES symptoms in MRI and the presence of seizures in the patients (*p* value = 0.016) [22].

These findings led to the conclusion that while the presence of the LFS and BG involvement on MRI scans is a specific finding associated with UE, their absence does not exclude the diagnosis. Therefore, a comprehensive approach incorporating clinical manifestations, laboratory test analyses, and imaging findings should be employed for the accurate diagnosis and management of UE.

Gray and white matter involvement in UE primarily affects the BG, thalami, midbrain, mesial temporal lobes, the parieto–occipital lobes, posterior–frontal cortex, and subcortical white matter [22,32,33].

Notably, this cortical involvement often presents alongside features suggestive of PRES, which are characterized by T2 hyperintense lesions commonly found in the subcortical white matter of the parieto–occipital regions on both sides [34,35,36,37,38].

To detect PRES lesions in the supratentorial brain regions, FLAIR imaging is considered more effective than T2WI [34].

## 4. Differential Diagnosis

The features of UE, akin to other metabolic encephalopathies, span from subtle executive dysfunction to coma. Early signs may include reduced attention, impaired construction and writing, executive dysfunction, behavioral changes, and sleep disturbances, which can escalate to an agitated delirium and coma. Hyperventilation may occur during periods of metabolic acidosis. Motor manifestations comprise generalized weakness, paratonia, multifocal myoclonus, action myoclonus, stimulus-sensitive myoclonus, tremor, and asterixis. Notably, myoclonus tends to be more pronounced in uremia compared to most other metabolic encephalopathies, often showing a response to clonazepam. Asterixis, described as periodic loss of muscle tone (i.e., negative myoclonus), may manifest as a flapping of the wrists with the arms outstretched and wrists hyperextended. Tetany, due to abnormal calcium homeostasis, may also occur. Seizures, typically generalized, tend to arise during the anuric or oligarch phase. Uremic coma, though now rare, typically presents with Kussmaul breathing associated with metabolic acidosis. In chronic UE, features include slowness of thought, headache, apathy, flattening of affect, inattention, and constructional impairment. Sleep disturbances and restless leg syndrome are common. Diffuse motor abnormalities such as tremor, myoclonus, and asterixis may be observed. While generalized convulsive seizures can occur in chronic UE, they tend to manifest at the terminal stage of the disease, often accompanied by stupor or coma [13,17,19,21,39].

Due to these considerations, UE presents a diagnostic challenge because its symptoms overlap with other neurological conditions. It can be confused with metabolic disorders like hepatic encephalopathy or diabetic crises and symptoms similar to those from infections such as meningitis.

Toxic and metabolic encephalopathy, which encompasses conditions such as carbon monoxide, cyanide, cocaine, opiate, and manganese intoxication, as well as Wilson disease, often target pallidal neurons selectively [40,41,42].

Globus pallidus is often involved in these conditions; however, even though UE involves the BG, the specific vulnerability of the globus pallidus remains uncertain in this condition, despite reports of cytotoxic edema in some UE cases [20].

In case of uncertainty, the use of DWI could be crucial, helping differentiate UE from other conditions with similar symptoms, such as metabolic disorders or infections. In particular, assessing the pattern and distribution of diffusion abnormalities, diagnosis could be more accurate, with subsequent more tailored treatment strategies [20,27].

Drug-induced neurological disturbances are also a concern, especially in patients with reduced kidney function who cannot efficiently clear medications. Structural brain problems like strokes or autoimmune disorders such as vasculitis may present similarly.

Another key condition for comparison is PRES, which shares many symptoms and imaging features with UE. Furthermore, various pathological studies have documented cytotoxic edema in PRES, which is characterized by fibrinoid necrosis and microinfarction. Additionally, there is a significant correlation between the signs of PRES on MRI and the occurrence of seizures in patients [22].

These studies have proposed a potential causal link between cytotoxic edema and the development of larger areas of vasogenic edema [43].

The importance of this study lies in its comprehensive approach to synthesizing the existing literature on MRI findings in UE. While previous studies have documented various aspects of cognitive changes and MRI abnormalities associated with uremic toxins, there has been a significant gap in correlating these findings with clinical manifestations and diagnostic challenges. This narrative review bridges that gap, offering several key contributions that enhance our understanding and management of UE.

**Figure 2:**
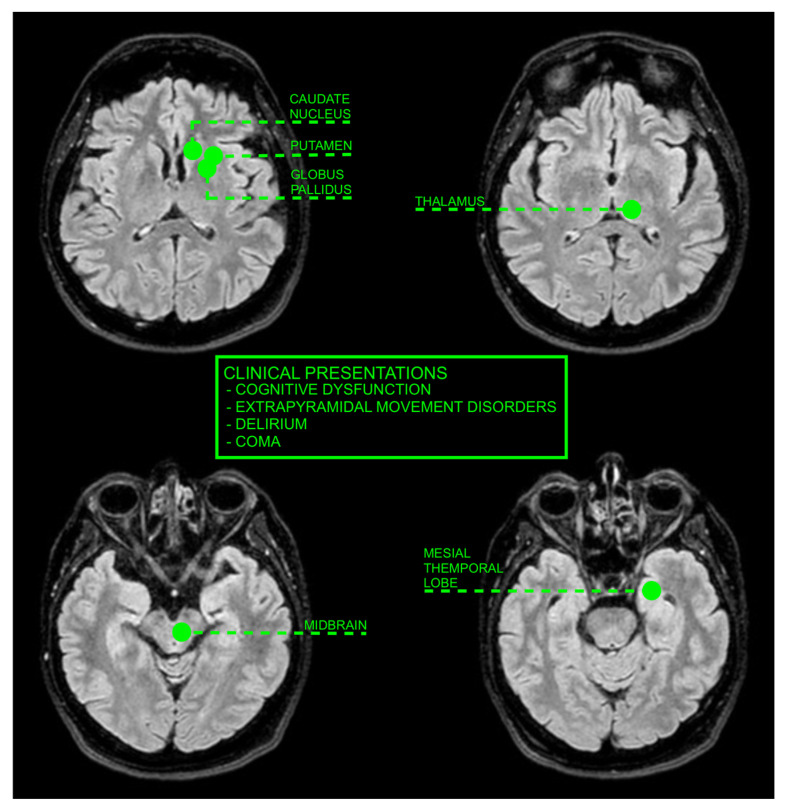
FLAIR images on the axial plane show the anatomical structures involved in UE and their main clinical presentations.

This study provides a detailed synthesis of MRI findings, categorizing them based on the affected brain regions and the type of edema. This categorization is crucial for radiologists and clinicians, as it aids in the differential diagnosis and accurate identification of UE. The review highlights specific MRI patterns, such as the LFS and BG involvement, which are instrumental in recognizing UE, especially in complex cases where other metabolic or toxic encephalopathies may present with similar symptoms. Another significant contribution of this study is its discussion on the differential diagnosis of UE. Given the overlap in symptoms and MRI findings between UE and other conditions such as PRES, hepatic encephalopathy, and drug-induced neurological disturbances, this review underscores the importance of a comprehensive diagnostic approach. By integrating clinical manifestations, laboratory test results, and imaging findings, clinicians can more accurately diagnose and manage UE, reducing the risk of misdiagnosis and ensuring better patient outcomes (Figure 2). Lastly, this study highlights the limitations of current research on UE and MRI. By identifying gaps such as the lack of long-term follow-up studies and the need for larger well-designed cohorts, the review sets the stage for future research directions. Addressing these limitations will be crucial for advancing our understanding of UE and improving diagnostic and therapeutic approaches.

## 5. Treatment

Using MRI is essential for ruling out other causes and guiding accurate diagnosis and appropriate treatment. Treatment of UE centers on addressing the root cause of kidney failure to mitigate the accumulation of toxins. Central to this approach is dialysis, including both hemodialysis and peritoneal dialysis, which plays a pivotal role in removing toxins and easing symptoms. Fine-tuning the dialysis protocol, coupled with medications to manage anemia and blood pressure, is essential for improving overall patient health. Improvements in the clinical status of patients with UE have been linked to the normalization of MRI changes, which is often due to the reversibility of renal failure, achieved through the abovementioned therapeutic interventions [20,27,44].

## 6. MRI and Study Limitations

While the utilization of MRI plays a pivotal role in the assessment of UE, certain limitations should be acknowledged. Firstly, MRI findings, including BG involvement and the LFS, while specific to UE, may not always be present, potentially leading to diagnostic challenges. Additionally, the absence of these specific MRI markers does not rule out the diagnosis of UE. This underscores the need for a comprehensive diagnostic approach that incorporates clinical manifestations, laboratory analyses, and imaging findings. Furthermore, while MRI provides valuable insights into UE pathology, it may not always offer a definitive diagnosis on its own. Interpretation of MRI findings requires careful consideration of other potential differential diagnoses, such as PRES, which shares overlapping imaging features with UE. Therefore, clinical correlation and integration with other diagnostic modalities are essential to accurately diagnose and manage UE. Moreover, while MRI sequences such as T1WI, T2WI, and FLAIR sequences offer valuable information about tissue characteristics and lesions, they may not always provide sufficient specificity to distinguish between different etiologies of brain pathology. Similarly, although DWI can help discriminate between vasogenic and cytotoxic edema, its interpretation may be subjective and influenced by various factors. Last but not least, the literature highlights several primary limitations in the study of UE. In fact, there is a scarcity of studies, and those available often involve small patient cohorts, compromising the generalizability of findings. Additionally, the absence of long-term clinical and imaging follow-up in these studies hampers the understanding of disease progression and treatment outcomes. These limitations underscore the need for larger well-designed studies with comprehensive long-term follow-up to elucidate the clinical and imaging characteristics of UE more accurately.

## 7. Conclusions

This literature review emphasizes MRI’s pivotal role in diagnosing UE highlighting its importance for early detection and distinguishing between ischemic and edematous brain lesions. MRI, combined with clinical assessments, is essential for effective UE management. Key MRI sequences such as T1WI, T2WI, FLAIR, and DWI provide detailed insights into brain tissue characteristics and types of brain edema. Although contrast-enhanced MRI is less useful, typical MRI findings in UE include brain edema in various regions. Accurate UE diagnosis and management require a comprehensive approach, integrating clinical, laboratory, and imaging data. Even though the recovery of neurological functions can vary, with some severe cases leading to irreversible damage, treatments like hemodialysis can reverse symptoms and MRI findings, underscoring the importance of timely intervention.

## Figures and Tables

**Table 1:** Clinical manifestations of uremic encephalopathy, categorized by severity from mild to severe and dependent on the rate of renal function decline.

Severity Level of Encephalopathy	eGFR	Clinical Manifestations
Early	40 to 60 mL/min	Fatigue, anorexia, nausea, insomnia, restlessness, decreased attention span, cognitive and memory impairments, difficulty managing ideas, apathy
Mild	30 to 39 mL/min	Similar to early stage but with increased severity; potential mild disorientation and subtle personality changes
Moderate	15 to 29 mL/min	Severe cognitive dysfunction (affecting memory, perception, abstraction), disorientation, slurred speech, disturbances in sleep patterns, mild to moderate deterioration in consciousness, delirium
Severe	Less than 15 mL/min	Severe cognitive dysfunction, disorientation, slurred speech, highly disturbed sleep patterns, significant deterioration in level of consciousness, delirium, bizarre behavior, multifocal myoclonus, asterixis, convulsions, psychosis (including visual hallucinations and delusions), catatonia, stupor, and in extreme cases, coma

**Table 2:** Summary of the articles included in this review.

Authors	Year	Country	Aim/Rationale	Patients (n°)	Conclusions
Kim et al. [18]	2016	Republic of Korea	Describe MR imaging findings of UE and correlate them with clinical conditions	10	Lentiform fork sign is a reliable early diagnostic indicator; lesions can be cytotoxic and/or vasogenic and may resolve post-dialysis.
Wang et al. [19]	2003	Taiwan	Clarify the clinical spectrum and possible pathophysiology of UE with BG involvement	6	BG lesions in uremic patients are associated with acute movement disorders and are not uncommon. The pathophysiology remains unclear.
Lee et al. [20]	2007	Republic of Korea	Investigate acute bilateral BG lesions in diabetic uremic patients	4	Bilateral BG lesions are primarily vasogenic, with some foci of cytotoxic edema. Lesions and symptoms are reversible post-hemodialysis.
Wang et al. [21]	1998	Multiple	Report on acute and subacute extrapyramidal movement disorders in uremic patients with BG lesions	3	UE with BG lesions leads to movement disorders and is linked to hypoperfusion and toxin vulnerability of the BG.
Sina et al. [22]	2022	Iran	Investigate MRI findings for diagnosing UE and evaluate their diagnostic usefulness	20	MRI findings, including white matter involvement and cerebral atrophy, are critical for diagnosing UE, alongside clinical and laboratory analyses.

Abbreviation: BG, basal ganglia; UE, uremic encephalopathy.
